# Glycosylated hemoglobin (HbA1c) levels and clinical outcomes in diabetic patients following coronary artery stenting

**DOI:** 10.1186/1475-2840-11-82

**Published:** 2012-07-17

**Authors:** Seyed Ebrahim Kassaian, Hamidreza Goodarzynejad, Mohammad Ali Boroumand, Mojtaba Salarifar, Farzad Masoudkabir, Mohammad Reza Mohajeri-Tehrani, Hamidreza Pourhoseini, Saeed Sadeghian, Narges Ramezanpour, Mohammad Alidoosti, Elham Hakki, Soheil Saadat, Ebrahim Nematipour

**Affiliations:** 1 Department of Cardiology, Tehran Heart Center, Tehran University of Medical Sciences, Tehran, Iran; 2 Research Department, Tehran Heart Center, Tehran University of Medical Sciences, Tehran, Iran; 3 Department of Clinical and Surgical Pathology, Tehran Heart Center, Tehran University of Medical Sciences, Tehran, Iran; 4 Students’ Scientific Research Center (SSRC), Tehran University of Medical Sciences, Tehran, Iran; 5 Endocrinology and Metabolism Research Center (EMRC), Shariati Hospital, Tehran University of Medical Sciences, Tehran, Iran; 6 Sina Trauma Research Center,Tehran University of Medical Sciences, Tehran, Iran; 7 Tehran Heart Center, 1411713138, Karegar Shomali St., Jalal al-Ahmad Cross, Tehran, Iran

**Keywords:** Diabetes mellitus, Percutaneous coronary intervention, Glycaemic control, Major adverse cardiovascular events

## Abstract

**Background:**

Diabetes has been shown to be independent predictor of restenosis after percutaneous coronary intervention (PCI). The aim of the present study was to investigate whether a pre- and post-procedural glycaemic control in diabetic patients was related to major advance cardiovascular events (MACE) during follow up.

**Methods:**

We evaluated 2884 consecutive patients including 2181 non-diabetic patients and 703 diabetics who underwent coronary stenting. Diabetes mellitus was defined as the fasting blood sugar concentration ≥ 126 mg/dL, or the use of an oral hypoglycemic agent or insulin at the time of admission. Diabetic patients were categorized into two groups based on their mean HbA1c levels for three measurements (at 0, 1, and 6 months following procedure): 291 (41.4%) diabetics with good glycaemic control (HbA1c ≤ 7%) and 412 (58.6%) diabetics with poor glycaemic control (HbA1c > 7%).

**Results:**

The adjusted risk of MACE in diabetic patients with poor glycaemic control (HbA1c > 7%) was 2.1 times of the risk in non-diabetics (adjusted HR = 2.1, 95% CI: 1.10 to 3.95, *p* = 0.02). However, the risk of MACE in diabetics with good glycaemic control (HbA1c ≤ 7%) was not significantly different from that of non-diabetics (adjusted HR = 1.33, 95% CI: 0.38 to 4.68, *p* = 0.66).

**Conclusions:**

Our data suggest that there is an association between good glycaemic control to obtain HbA1c levels ≤7% (both pre-procedural glycaemic control and post-procedural) with a better clinical outcome after PCI.

## Background

Despite recent advances in medical management and coronary revascularization, cardiovascular disease accounts for about 75% of all hospital admissions and 80% of deaths in diabetic patients [1]. Although the introduction of drug-eluting stents has reduced the rates of restenosis and clinical events after percutaneous coronary intervention (PCI), since the diabetes mellitus has been proved to be a strong risk factor for in-stent restenosis [2-4], restenosis after stent implantation remains the “Achilles’ heel” of PCI [5], and patients with diabetes still have poorer clinical outcomes compared with non-diabetics [6-10].

The higher rates of restenosis in diabetic patients might be partly explained by exaggerated neointimal proliferation after stent implantation due to hyperinsulinemic state of diabetes [11]. Glycosylated hemoglobin (HbA1c) is reflective of mean ambient fasting and postprandial plasma glucose levels over the preceding 2 to 3 months [12,13]. There is consistent evidence that optimal glycaemic control (defined as HbA1c ≤ 7%) results in a lower incidence of microvascular complications in both type 1 and type 2 diabetes mellitus [14]. However, the corollary that optimal glycaemic control in diabetic patients would lead to a similar improvement in clinical outcome of PCI has not been extensively investigated. While there are conflicting data regarding the effect of preprocedural glycaemic control on outcome of PCI, there is limited data about the impact of post-procedural glycaemic control, beginning at the time of PCI and continuing afterwards, on incidence of MACEs after PCI.

In this study, we sought to investigate whether a pre- and post-procedural glycaemic control in diabetic patients, as reflected by mean plasma HbA1c levels prior to and 1 and 6 months after elective coronary stenting, was related to major advance cardiovascular events (MACE) during 1-year follow up.

## Methods

### Study population

**Figure 1:**
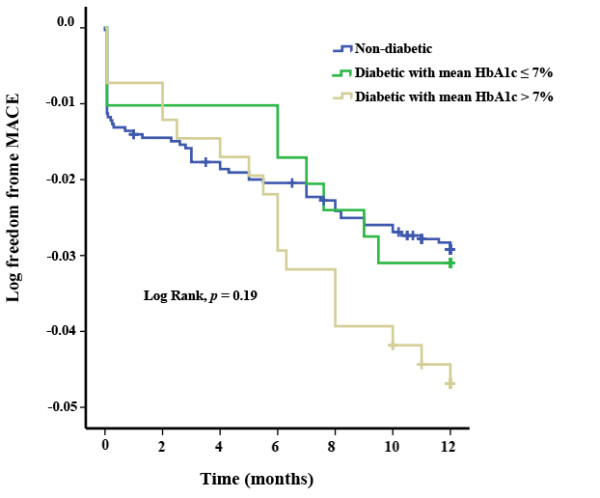
**Flow diagram of patient recruitment and follow-up.** HbA1c, glycosylated hemoglobin.

Between October 2007 and December 2009, all consecutive patients scheduled for elective PCI at the cardiac catheterization laboratory of our center were enrolled in this prospective cohort study (Figure 1). During this period angioplasty procedure were performed in 3964 patients. Patients requiring non-elective procedures for acute coronary syndromes (n = 869), and patients who refused to participate in the study (n = 102) were excluded. The remaining 2993 patients were compatible with our selection criteria. The study protocol was approved by the ethics committee of Tehran University of Medical Sciences. Written informed consents were obtained from all participants.

### Coronary procedures and adjunctive antiplatelet therapy

PCI and intracoronary stent implantation were performed according to current guidelines and using standard percutaneous techniques. Choosing the type of stent was at the discretion of the operator and each operator relied on his own judgment to assess stent expansion. All patients were on aspirin and received a 5,000–10,000 unit bolus unfractionated heparin in order to achieve an activated clotting time >250 sec. Patients also received 300–600 mg oral clopidogrel initiated either before or in the catheterization laboratory at the discretion of the operator, and continued at the dose of 75 mg/day for at least 1 month in bare metal and 12 months in drug eluting stents. GP IIb/IIIa antagonists were used on discretion of the operator.

### Biochemical analyses

After 10 h overnight fasting, peripheral venous blood specimens were obtained from participants via an antecubital vein. Fasting plasma glucose (FPG) was measured by a hexokinase enzyme method. HbA1c was measured by an immunoturbidimetric method using automatic analyzer (COBAS INTEGRA 400, Roche Diagnostics GmbH, Mannheim, Germany). Total cholesterol, HDL-cholesterol, and triglycerides levels were assessed using enzyme-colorimetric tests; LDL cholesterol was estimated based on Friedewald’s formula. LDL-cholesterol was not calculated if the serum triglyceride level was more than 400 mg/dl. Assay performance was monitored every 50 tests, using the lipid control serum available commercial kit. Baseline blood sampling (before cardiac catheterization) for HbA1c repeated at 1 and 6 months after procedure.

### Study endpoints and definitions

Diabetes mellitus was defined as the fasting blood sugar concentration ≥ 126 mg/dL, or the use of an oral hypoglycemic agent or insulin at the time of admission. The HbA1c levels before coronary stenting, and 1 and 6 months after the procedure was measured for diabetic patients and the mean of the 3 measurements was considered for assessing the patients’ glycaemic control. “Good-control group” was defined as diabetic patients with mean HbA1c ≤ 7%, “poor-control group” was defined as diabetic patients with mean HbA1c > 7%. Procedural success was defined as Thrombolysis In Myocardial Infarction Flow (TIMI) grade 3 with a residual stenosis < 10%. Myocardial infarction (MI) was defined as the acute-onset chest pain and/or typical modification on electrocardiogram (ST- or T-wave modification or new left bundle branch block) and an elevation of creatine kinase to > 3 times of the upper reference limit. Target vessel revascularization (TVR) was characterized by ischemia-driven percutaneous or surgical revascularization of the treated vessel. Target lesion revascularization (TLR) by PCI was defined as treatment of a lesion in the stent or within 5 mm of the stent borders. The primary end point of the present study was 12-month cumulative MACE, defined as death, non-fatal MI, and the need for TVR.

### Patient follow-up

The data on the early outcomes and occurrence of death, new non-fatal MI, need for CABG, subsequent need for repeat PCI in all groups were recorded. Follow up visits were scheduled at 1, 6, and 12 months after procedure conducted by clinic visits or if the patients did not attend clinics for the scheduled visits performed by telephone interviews. For the telephone follow-up interviews, at least five attempts were made to contact participants or their first-degree relatives. If telephone interviews were unsuccessful, the participants were contacted by mail using their home address.

Of a total of 2993 patients compatible with our selection criteria, 55 (51 in non-diabetic group, 3 in controlled group and 1 in uncontrolled group) patients were lost at the 1-month and 54 (47 in non-diabetic group, 4 in good-control group and 3 in poor-control group) patients at the 6- month follow-up.

Revascularization of the target vessel was considered to have been prompted by ischemia if there was evidence of angina. If the patients did not have angina symptoms, a functional stress test was performed within 12 months after the procedure to reveal silent ischemia. Repeat cardiac catheterization was performed for recurrent symptoms or objective evidence of ischemia with provocative testing. Routine angiographic follow-up was not undertaken. One-year clinical follow-up rate was 96.1%.

### Statistical analysis

The Kolmogorov-Smirnov test was applied to examine normal distribution. Continuous variables are expressed as mean ± SD and were compared among non-diabetic, diabetic controlled, and diabetic uncontrolled groups by analysis of variance (ANOVA) followed by Scheffe’s post-hoc test for pairwise comparisons. Categorical variables were compared using a chi-square test, and were presented as absolute frequencies with percentages. Event-free survival curves were constructed using the Kaplan-Meier method and compared using log-rank test. Individuals were censored at the first cardiovascular event. Cox multivariate analyses were used to determine independent predictors of MACE. Variables were entered into the model based on their statistical significance in univariable analyses (entering criterion p ≤ 0.20) as well as their clinical significance. To exclude the impact of HbA1c level fluctuation during the follow-up period and appropriately define the patients’ group, landmark analyses were performed at the landmark times of 1-month and 6-month [15]. For all analysis, the statistical package SPSS version 15.0 for windows (SPSS Inc, Chicago, Illinois, USA) was used. All p values were 2-tailed with significance defined as *p* ≤ 0.05.

## Results

Among 2884 patients (mean age ± SD, 57.7 ± 10.6 years; 70.1% men) who entered in the analysis for assessment of the outcomes, 2181 (75.6%) were non-diabetic and 703 (24.4%) were diabetic. Of the 703 diabetic patients, 291 (41.4%) were controlled and 412 (58.6%) were uncontrolled. The baseline clinical and laboratory characteristics of the study patients are presented in Table 1. Diabetic patients were older and were less likely to be male as compared to non-diabetics. The prevalence of current smoking and statin-use was also lower in diabetic patients compared with non-diabetic patients; however, patients with diabetes had higher prevalence of hypertension, hyperlipidemia, and ACEIs/ARBs consumption as well as greater body mass index (BMI) and waist circumference (WC). Diabetic patients with good glycaemic control were older and less often female than uncontrolled diabetics, and less commonly treated with insulin and ACEIs/ARBs.

**Table 1:** Baseline clinical and pharmacological characteristics of the study patients

	**Non-Diabetic (n = 2181)**	**Good-control DM (n = 291)**	**Poor-control DM (n = 412)**	P-value^*^	**P-value**^**†**^	**P-value**^**‡**^
Age (year)	57.4 ± 10.8	59.9 ± 9.9	58.0 ± 9.8	<0.0001	0.27	0.009
Male sex	1653 (75.8)	165 (56.7)	203 (49.3)	<0.0001	<0.0001	0.07
Hypertension	1006 (46.1)	172 (59.1)	262 (63.6)	<0.0001	<0.0001	0.35
Hyperlipidemia	1318 (61.5)	224 (77.8)	328 (79.6)	<0.0001	<0.0001	0.58
Current smoking	1026 (47.0)	79 (27.1)	106 (25.7)	<0.0001	<0.0001	0.86
Family history of CAD	502 (23.1)	63 (22.1)	89 (21.6)	0.77	0.45	0.78
BMI (kg/m^2^)	27.3 ± 4.25	28.3 ± 4.1	28.2 ± 4.5	<0.0001	< 0.0001	0.85
WC (cm)	99.4 ± 9.7	101.9 ± 9.0	102.3 ± 10.1	<0.0001	< 0.0001	0.61
EF < 30%	68 (4.3)	7 (3.5)	17 (5.9)	0.71	0.18	0.21
Medications						
Insulin	0 (0)	17 (6.0)	69 (17.0)	<0.0001	<0.0001	<0.0001
OHA	0 (0)	84 (28.9)	171 (41.5)	<0.0001	<0.0001	0.001
ACEIs/ARBs	1295 (59.4)	180 (61.9)	288 (69.9)	0.35	<0.0001	0.04
Statins	1849 (84.8)	255 (87.6)	362 (87.9)	0.121	0.06	0.912
Beta-blockers	1915 (87.8)	244 (83.8)	355 (86.2)	0.11	0.55	0.34

Data are presented as mean ± SD or n (%).

* Non-diabetic patients versus good-control (diabetic patients with HbA1c ≤ 7%).

† Non-diabetic patients versus poor-control (diabetic patients with HbA1c > 7%).

‡ Good-control versus poor-control diabetic patients.

ACEIs, angiotensin converting enzyme inhibitors; ARBs, angiotensin receptor blockers; BMI, body mass index; CAD, coronary artery disease; DM, diabetes mellitus; EF, ejection fraction; OHA, oral hypoglyceamic agent; WC, waist circumference.

As seen in Table 2, Diabetic patients showed higher levels of TG and FBS. Diabetic controlled group as compared to uncontrolled group had significantly lower levels of TG and HbA1c.

**Table 2:** Serum biochemistry profile of the study patients

	**Non-Diabetic (n = 2181)**	**Good-control DM (n = 291)**	**Poor-control DM (n = 412)**	P-value^*^	**P-value**^**†**^	**P-value**^**‡**^
LDL-C (mmol/l)	2.52 ± 1.02	2.43 ± 0.92	2.51 ± 1.15	0.12	0.80	0.33
HDL-C (mmol/l)	1.05 ± 0.28	1.05 ± 0.28	1.08 ± 0.56	0.95	0.27	0.36
TCH (mmol/l)	4.38 ± 1.18	4.28 ± 1.16	4.50 ± 1.35	0.17	0.10	0.02
TG (mmol/l)	1.90 ± 1.10	1.93 ± 1.09	2.15 ± 1.17	0.68	<0.0001	0.01
FPG (mmol/l)	5.34 ± 0.68	7.07 ± 2.14	10.19 ± 3.89	<0.0001	< 0.0001	< 0.0001
Mean HbA1c (%)	-	6.6 ± 0.7	8.8 ± 1.3	–	–	< 0.0001
Cr (μmol/l)	100.8 ± 27.6	101.3 ± 60.8	99.8 ± 32.5	0.88	0.58	0.68

Data are presented as mean ± SD.

* Non-diabetic patients versus good-control (diabetic patients with HbA1c ≤ 7%).

† Non-diabetic patients versus poor-control (diabetic patients with HbA1c > 7%).

‡ Good-control versus poor-control diabetic patients.

FPG, fasting plasma glucose; HDL-C, high-density lipoprotein cholesterol; LDL-C, low-density lipoprotein cholesterol; TCH, total cholesterol; TG, triglycerides; Cr, creatinine.

The cardiac catheterization data of the study patients are summarized in Table 3. Diabetic patients had higher prevalence of 3-vessel disease and were more treated with drug-eluting stent. There were no significant differences between the diabetic and non-diabetic patients regarding the rest of clinical, laboratory and cardiac catheterization parameters. Also, no significant difference was observed between diabetic controlled and uncontrolled with respect to cardiac catheterization parameters.

**Table 3:** Cardiac catheterization data

	**Non-Diabetic (n = 2181)**	**Good-control DM (n = 291)**	**Poor-control DM (n = 412)**	P-value^*^	**P-value**^†^	**P-value**^**‡**^
Type C lesion	1458 (66.9)	203 (69.8)	280 (68.0)	0.33	0.69	0.62
CTO	78 (3.6)	6 (2.1)	11 (2.7)	0.23	0.46	0.63
3-vessel disease	358 (16.8)	57 (20.2)	102 (25.1)	0.18	<0.0001	0.12
RVD < 3 mm	1092 (50.1)	165 (56.7)	236 (57.3)	0.04	0.006	0.88
SSL > 30 mm	896 (41.1)	128 (44.0)	174 (42.2)	0.35	0.66	0.70
CR	1312 (60.2)	164 (56.4)	213 (51.7)	0.21	0.001	0.22
Drug-eluting stent	1159 (54.6)	192 (67.1)	272 (66.8)	<0.0001	<0.0001	0.86
Number of stents	1.3 ± 0.6	1.3 ± 0.6	1.3 ± 0.5	0.33	0.41	0.81

Data are presented as mean ± SD or n (%).

* Non-diabetic patients versus good-control (diabetic patients with HbA1c ≤ 7%).

† Non-diabetic patients versus poor-control (diabetic patients with HbA1c > 7%).

‡ Good-control versus poor-control diabetic patients.

CR, complete revascularization CTO, chronic total occlusion; RVD, reference vessel diameter; SSL, sum of the stents’ length.

**Figure 2:**
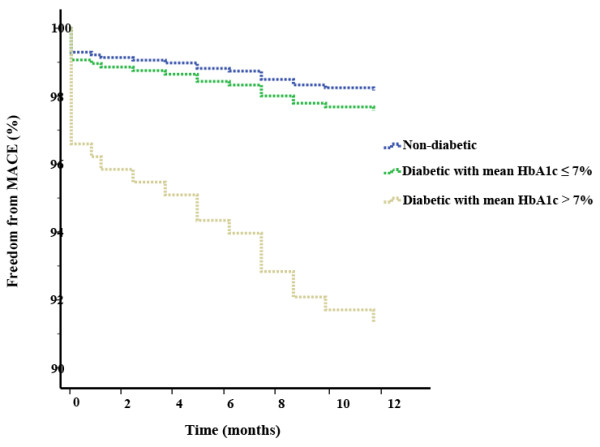
Kaplan-Meier event-free survival curves for freedom of MACE in the 3 groups of non-diabetic patients, diabetics with good control (mean HbA1c ≤ 7%) and diabetics with poor control (mean HbA1c > 7%).

During the 12-months follow-up in the entire population, 95 (3.3%) MACEs comprised of 40 (1.4%) TVR, 36 (1.2%) non-fatal MI, and 19 (0.6%) cardiovascular mortality were indexed. Although there was significant difference between the poor-controlled diabetic patients and non-diabetics with respect to rate of TVR (3.2% vs. 1.1%, respectively, *p* = 0.002), the rate was similar in good-controlled diabetics and non-diabetics (1.4% vs. 1.1%, *p* = 0.54). There were no statistically significant differences among the groups with respect to non-fatal MI, cardiovascular mortality, in-hospital MACEs and total-MACEs (Table 4). Figure 2 demonstrates freedom-from-MACE survival curves in the three groups of non-diabetics, good-controlled diabetics, and poor-controlled diabetics.

**Table 4:** Follow-up data

	**Non-Diabetic (n = 2181)**	**Good-control DM (n = 291)**	**Poor-control DM (n = 412)**	P-value^*^	**P-value**^**†**^	**P-value**^**‡**^
Total MACE	65 (3.0)	9 (3.1)	21 (5.1)	0.85	0.09	0.33
In-hospital MACE	27 (1.2)	3 (1.0)	3 (0.7)	1.00	0.79	0.70
TVR	23 (1.1)	4 (1.4)	13 (3.2)	0.55	0.002	0.14
TLR	9 (0.4)	3 (1.0)	4 (1.0)	0.16	0.13	1.00
CABG	10 (0.4)	0 (0)	6 (1.4)	0.62	0.03	0.04
Non-fatal MI	26 (1.1)	4 (1.4)	6 (1.4)	0.77	0.62	1.00
All-cause mortality	25 (1.1)	1 (0.3)	4 (1.0)	0.35	1.00	0.41
Cardiac death	16 (0.7)	1 (0.3)	2 (0.5)	0.71	1.00	1.00
Non-cardiac death	9 (0.4)	0 (0)	2 (0.5)	0.61	0.69	0.51

Data are presented as n (%).

* Non-diabetic patients versus good-control (diabetic patients with HbA1c ≤ 7%).

† Non-diabetic patients versus poor-control (diabetic patients with HbA1c > 7%).

‡ Good-control versus poor-control diabetic patients.

CABG, coronary artery bypass grafting surgery; MACE, major adverse cardiovascular events; MI, myocardial infarction; TLR, target lesion revascularization; TVR, target vessel revascularization.

**Figure 3:**
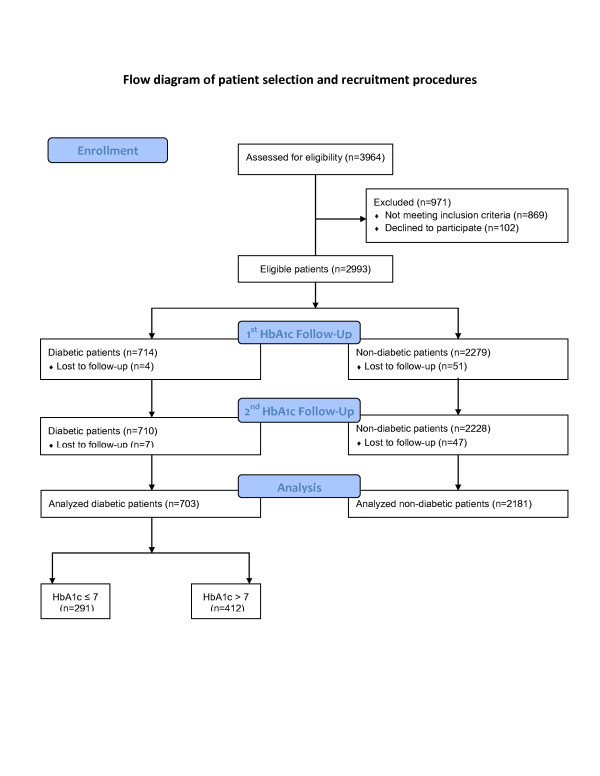
Cox-adjusted event-free survival curves for freedom of MACE in the 3 groups after adjustment for potential confounders including age, sex, hypertension, hyperlipidemia, current smoking, waist circumference, insulin therapy, ACEIs/ARBs use, statin use, and 3-vessel disease.

Table 5 presents uni- and multi-variable Cox regression analysis for predictors of MACE. After adjustment for potential confounders, the risk of MACE in diabetic uncontrolled patients (HbA1c > 7%) was 2.1 times of the risk in non-diabetic patients (adjusted HR = 2.09; 95% CI, 1.10 to 3.95; *p* = 0.02). The confounders included age, sex, hypertension, hyperlipidemia, current smoking, waist circumference, insulin therapy, ACEIs/ARBs use, statin use, 3-vessel disease, and complete revascularization. However, the risk of MACE in diabetic patients with good glycaemic control (HbA1c ≤ 7%) was not significantly different from that of non-diabetic patients (adjusted HR = 1.48; 95% CI, 0.68 to 3.21; *p* = 0.32). Figure 3 shows freedom from MACE curves in the three groups after adjustment for potential confounders.

**Table 5:** Cox regression analysis for predictors of major adverse cardiovascular events at 12 months

	**Univariable analysis**			**Multivariable analysis**		
	**Crude HR**	**95% CI**	***P***	**Adjusted HR**	**95% CI**	***P***
Age (year)	1.01	0.99 to 1.03	0.15	1.01	0.98 to1.03	0.53
Male sex	1.14	0.72 to 1.83	0.57	1.80	0.96 to 3.38	0.07
Hypertension	1.02	0.68 to 1.53	0.92	0.99	0.60 to 1.65	0.98
Hyperlipidemia	0.86	0.56 to 1.31	0.48	0.99	0.59 to 1.65	0.97
Current smoking	0.65	0.42 to 1.00	0.06	0.60	0.35 to 1.03	0.07
Waist circumference	0.98	0.95 to1.00	0.04	0.97	0.95 to 1.00	0.03
Insulin	1.82	0.74 to 4.47	0.19	1.09	0.32 to 3.73	0.89
ACEIs/ARBs use	1.12	0.73 to 1.70	0.61	0.97	0.59 to 1.59	0.44
Statin use	0.91	0.52 to 1.62	0.76	0.81	0.42 to 1.56	0.53
3-vessel disease	1.35	0.83 to 2.20	0.23	1.15	0.61 to 2.12	0.67
Drug-eluting stent	0.68	0.45 to 1.03	0.07	0.54	0.33 to 0.87	0.01
CR	0.63	0.41 to 0.96	0.03	0.75	0.43 to 1.29	0.30
Group						
Non-Diabetics	1*	–	–	1*	–	–
Good-control diabetics ^1^	1.06	0.53 to 2.12	0.88	1.48	0.68 to 3.21	0.32
Poor-control diabetics ^2^	1.59	0.96 to 2.67	0.07	2.09	1.10 to 3.95	0.02

* Reference category.

ACEIs, angiotensin converting enzyme inhibitors; ARBs, angiotensin receptor blockers; CI, confidence interval; HR, hazard ratio; WC, waist circumference; CR, complete revascularization.

^1^ Diabetic patient with HbA1c ≤ 7%.

^2^ Diabetic patient with HbA1c > 7%.

We defined the patients’ group based on the average of 3-times measurements of HbA1c levels, before PCI and 1 and 6 months after PCI. However, a total of 11 diabetic patients were lost to follow-up by 6 months of follow-up. Furthermore, HbA1c level fluctuated around 7.0% over time leading to crossing over between good-control and poor-control group classifications. In order to exclude the impact of HbA1c level fluctuation around 7.0% during the follow-up period and appropriately define the patients’ group, we employed 1-month and 6-month landmark analyses to examine the association of post-procedural glycemic control and 1-year clinical MACE of diabetic patients undergoing PCI at the landmark times. The results are summarized in Table 6.

**Table 6:** Multivariable Cox regression model for detecting the independent effect of post-procedural diabetic control on major adverse cardiovascular events (MACE) at 12 months

	**Odds ratio**	**95% confidence interval**	**P value**
**At Baseline**			
Non-diabetics (n = 2181)	1*	–	–
Good-control diabetics ^1^ (n = 291)	1.54	0.74 to 3.19	0.250
Poor-control diabetics ^2^ (n = 412)	1.98	1.06 to 3.68	0.032
**1-month landmark analysis**			
Non-diabetics (n = 2147)	1*	–	–
Good-control diabetics ^1^ (n = 295)	2.02	0.80 to 5.09	0.136
Poor-control diabetics ^2^ (n = 402)	3.25	1.57 to 6.71	0.001
**6-month landmark analysis**			
Non-diabetics (n = 2133)	1*	–	–
Good-control diabetics ^1^ (n = 256)	1.40	0.60 to 7.90	0.236
Poor-control diabetics ^2^ (n = 430)	4.10	1.04 to 7.81	0.043

*Reference group.

^1^ Diabetic patient with HbA1c ≤ 7%.

^2^ Diabetic patient with HbA1c > 7%.

## Discussion

In-stent restenosis (ISR) is mainly caused by the effects of vascular smooth muscle cell proliferation (neointimal hyperplasia) and migration. As part of mechanical injury response, immediately after stent placement, endothelium damage and the deposition of a layer of platelets and fibrin occur at the site of injury [16]. ISR also occurs lately over several months at the location around stent struts by a chronic inflammatory phase. Neointima increases up to three months after stenting, with little change to six months, and a gradual decrease thereafter [17]. Thus, the time frame of first 6 months after stent implantation was used in this study as follow-up to look at glycemic control.

An “exaggerated” vascular proliferation is observed in patients with diabetes mellitus. In diabetic animals, hyperinsulinemia rather than hyperglycemia per se, appears to be important in determining the exaggerated neointimal hyperplasia after balloon angioplasty [18]. In order to assess this topic in human, we analyzed for the effect of the post-procedural glycaemic control in the first six months after PCI on occurrence of MACE.

The main finding of our study was that glyceamic control to be significantly associated with 1-year outcome in diabetic patients undergoing elective PCI with stent implantation. We observed that diabetics with poor glycaemic control are at 2.1 times more risk of developing MACE while good-controlled diabetics showed rates of adverse clinical events comparable to those of non-diabetic patients. The higher MACE rate in the poor-control group was mostly driven by a higher rate of TVR.

Our findings are in agreement with several previous studies reporting increased rates of MACE following PCI in uncontrolled diabetic patients [19-23]. Among studies two previous investigators [19,20] examined the effect of glycaemic control on need for TVR in diabetic patients undergoing elective PCI. They observed lower rates of TVR, cardiac rehospitalization and recurrent angina in optimally-controlled (HbA1c ≤ 7%) diabetic patients. comparable rates of adverse events in optimally-controlled diabetics and non-diabetics were also observed. A historical cohort study on 206 diabetic patients with drug-eluting stent implantation showed that pre-procedural HbA1c level is an independent predictor of MACE [21]. It is also demonstrated that a HbA1c concentration of 6% to 7% is associated with a significantly higher risk of MACEs, TVR, and cardiovascular mortality following elective PCI in nondiabetic patients [24].

However, conflicting findings exist on the impacts of intensive glucose control with aggressive HbA1c goals on cardiovascular events [25-27]. In a 2-year follow-up study [28], the incidence of cardiovascular events was statistically similarly increased after acute myocardial infarction (AMI) in known diabetics and newly diagnosed diabetics compared with non-diabetic patients including IGT patients. However, when baseline characteristics were compared between the two groups, the level of HbA1c of newly diagnosed diabetics was significantly lower than that of known diabetic group (5.7 ± 0.4 mg/dL vs. 8.1 ± 1.5 mg/dL, p < 0.05). There are several previously published studies reporting that pre-procedural HbA1c levels are not predictive of cardiovascular events in diabetic patients following successful PCI [29-33]. In a recently published study on 952 diabetic patients undergoing PCI with stent implantation, no significant relationship was observed between pre-procedural HbA1c levels and patients’ outcome[33]. The investigators attributed such conflicting finding, at least in part, to high rate of drug-eluting stent use in their study (70%). The use of drug-eluting stent in our study (about 66% in diabetic patients and 53% in non-diabetics) was similar to that study [33]; however, we observed that pre- and post-procedural glycaemic control predicts MACE following PCI even after adjustment for potential confounding effect of drug-eluting stent implantation.

Observational data relating uncontrolled diabetes to higher rates of cardiovascular events in this group of patients encouraged the researchers to assess the effect of therapies that improve glycemic control on cardiovascular risk. Clinical trials have already demonstrated that therapies that improve glycemic control decrease the risk of microvascular disease, including retinopathy, nephropathy, and neuropathy [34]. However, trials attempting to decrease macrovascular events have been unsuccessful; in the Action to Control Cardiovascular Risk in Diabetes (ACCORD) [35], Action in Diabetes and Vascular Disease: Preterax and Diamicron Modified Release Controlled Evaluation (ADVANCE) [36], and Veterans Affairs Diabetes Trial (VADT) studies [37], improved glycemic control showed no reduction in the rate of cardiovascular events and in ACCORD trial [35], even it was associated with increased risks of death from any cause and death from cardiovascular events.

Ike and colleagues [38] have recently published a study on the effect of glycaemic control after PCI in patients with pre-procedural uncontrolled diabetes (HbA1c ≥ 6.9) and observed that glycemic control started at PCI and continued afterwards for approximately 300 days was not associated with improved clinical MACE at follow-up. The authors suggested that a so-called “metabolic memory (legacy) effect” which is a complex of MACE-increasing factors due to chronic hyperglycaemia might have adversely affected the clinical outcome in all diabetic patients with pre- procedural impaired glycaemic control irrespective of their post-procedural glycaemic control. However, this study was not a prospective cohort study and one cannot exclude the possibility that the clinical outcome may have been influenced for this reason.

The higher rates of MACE in diabetic patients with worse glycaemic control may have several explanations: There is evidence that chronic hyperglycemia induces vascular endothelial cell damage, with resultant vasomotor dysfunction, excessive extracellular matrix formation, and increased cellular proliferation. Hyperinsulinemia has been widely proposed as a predisposing factor for stent restenosis in diabetic patients [39], and concerns have been raised over the management of diabetes with exogenously administered insulin, as it may accelerate progression of CAD through its atherogenic mechanisms [40,41]. Currently data regarding the impact of insulin therapy on restenosis after PCI are controversial [42-44]. While Abizaid et al. [45] found an increased rate of TLR in insulin-treated diabetic patients compared with non-diabetic patients, others [46,47] demonstrated no significant difference in restenosis rates between the two groups. In our study there was no difference between the insulin-treated and non-insulin-treated diabetics with respect to TVR and MACE, and in multivariate Cox regression analysis insulin usage was not an independent predictor for MACE.

The novel aspect of our study was that in addition to pre-procedural HbA1c levels, we measured the post-procedural circulating HbA1c concentrations at 1 and 6 months after PCI. Hence, it not only reflects the ambient glycaemic control 2–3 months before procedure, but also surrogates the post-procedural glycaemic control in the first six months after PCI.

It is also notable that in this study we observed that current cigarette smoking and WC were tended to be predictors for better PCI outcome. Current smoking showed a protective effect on outcome of PCI in univariable analysis but it was not found to be an independent predictor for outcome after controlling for other covariates (Adjusted HR: 0.60, 95% CI: 0.35 to 1.03, p = 0.07). Waist circumference however showed statistically significant and independent protective effect on outcome of PCI in multivariable model. Although we don’t know the exact reason of such observation in our study, there is conflicting data regarding the effect of obesity on outcome of PCI [48], and a more optimal medical treatment in the obese group could explain the observed better outcome (“obesity paradox”) in these patients [49].

### Limitations

There are several potential limitations in our study that needs to be mentioned. First, in this study routine angiographic follow-up was not performed, and thus absolute restenosis rates could not be reported. Second, this was a single-center experience and larger multi-center studies should confirm our findings. Finally, although our method of assessment, protocol for calibrating HbA1c levels, and guidelines of reporting ensures high accuracy, HbA1c measurement error might be still a concern especially because this index was not derived from repeated measurements over time.

## Conclusions

In conclusion, these data suggest that good glycaemic control to obtain HbA1c levels ≤7% in diabetic patients undergoing coronary artery stenting may be beneficial in reducing the risk of restenosis and in improvement of the clinical outcome after PCI at 1-year follow-up.

## Competing interests

The authors declare that they have no competing interests.

### Authors’ contributions

SEK participated in study design, have revisited critically the manuscript for important intellectual content, and given final approval of the version to be published. MAB, MS, MRM, HP, SS, NR, MA, EH, and EN have made substantial contributions to conception and design, or acquisition of data, or analysis and interpretation of data. SS participated in the design of the study and supervised the statistical analysis. FM performed the statistical analysis and involved in drafting the manuscript. HG participated in study design and coordination and helped to draft the manuscript. All authors read and approved the final manuscript.
